# Hidden blood loss estimation using perioperative hemoglobin drop: agreement with the Sehat formula

**DOI:** 10.1186/s12893-026-03825-z

**Published:** 2026-05-20

**Authors:** Nikolai Ramadanov, Maximilian Heinz, Robert Prill, Dakota Fuchs, Roland Becker

**Affiliations:** 1 Center of Orthopaedics and Traumatology, Brandenburg Medical School, University Hospital Brandenburg, Brandenburg/Havel, Germany; 2 Faculty of Health Science Brandenburg, Brandenburg Medical School Theodor Fontane, Brandenburg/Havel, Germany

**Keywords:** Total hip arthroplasty, Hidden blood loss, Hemoglobin drop, Patient blood management, Perioperative bleeding

## Abstract

**Background:**

Perioperative hemoglobin drop is widely used as a surrogate marker for blood loss and transfusion risk across surgical disciplines. However, its agreement with dilution-based estimates of hidden blood loss remains insufficiently validated. As both approaches represent indirect estimators rather than a reference standard, this study evaluates agreement rather than validation.

**Methods:**

This retrospective registry-based study included 828 patients undergoing elective primary total hip arthroplasty between 2016 and 2023. Hemoglobin drop was defined as the difference between preoperative hemoglobin and the lowest postoperative value within 48 h. Hidden blood loss was calculated using the Gross–Sehat method with blood volume estimation according to Nadler. Agreement between hemoglobin drop and hidden blood loss was assessed using Bland–Altman analysis and intraclass correlation coefficients. Discriminative performance for identifying patients with high hidden blood loss (upper quartile) was evaluated using receiver operating characteristic analysis.

**Results:**

Mean perioperative hemoglobin drop was 33.5 ± 12.3 g/L. Mean hidden blood loss was 854.9 mL, accounting for the majority of total blood loss. Agreement between hemoglobin drop and hidden blood loss was limited, with wide limits of agreement on Bland–Altman analysis. Although a positive association was observed, substantial interindividual variability persisted, particularly at higher blood loss levels. Hemoglobin drop demonstrated only moderate discriminative performance for identifying patients with high hidden blood loss.

**Conclusion:**

Perioperative hemoglobin drop shows only moderate agreement with dilution-based estimates of hidden blood loss and cannot reliably replace formal blood loss calculations at the individual patient level. These findings have implications for perioperative blood management and caution against reliance on hemoglobin change alone for blood loss assessment.

**Level of Evidence (LoE):**

Level III - retrospective cohort study / registry-based observational study.

**Supplementary Information:**

The online version contains supplementary material available at 10.1186/s12893-026-03825-z.

## Introduction

Hidden blood loss (HBL) refers to perioperative blood loss that is neither directly measured intraoperatively nor captured through postoperative drainage. In elective total hip arthroplasty (THA), HBL can be substantial, contributing to postoperative anemia, increased transfusion requirements, delayed mobilization, and higher morbidity [1]. Reliable quantification of HBL is therefore essential for effective perioperative management and informed transfusion strategies.

The concept of HBL was first introduced by Sehat et al., who demonstrated that visible blood loss markedly underestimates total perioperative loss, with hidden losses accounting for approximately one-quarter to one-third of the total in both THA and total knee arthroplasty (TKA) [2]. Subsequent studies confirmed these findings and identified several patient- and surgery-specific predictors of HBL, such as body mass index, age, sex, surgical approach, and incision length [3, 4]. Recognition of these determinants supports early identification of patients at risk for clinically significant perioperative anemia.

Accurate assessment of total and hidden blood loss, however, remains methodologically challenging. Formula-based approaches incorporating patient blood volume (PBV) and perioperative changes in hemoglobin (Hb) or hematocrit are commonly used, but these methods require multiple laboratory measurements and rely on assumptions regarding fluid shifts and transfusions [5, 6]. Simplified approaches, particularly those based solely on perioperative Hb drop, have been proposed as practical bedside tools, with emerging evidence suggesting reasonable agreement with more complex formulas [7, 8].

Blood-management strategies, most notably the widespread adoption of tranexamic acid (TXA) and standardized perioperative protocols, have effectively reduced both visible and hidden blood loss in THA and lowered transfusion rates without increasing thromboembolic risk [9]. In this contemporary setting, accurate interpretation of commonly available clinical parameters, such as perioperative hemoglobin changes, remains essential for guiding perioperative decision-making [10].

Despite these advances, the clinical interpretation of perioperative hemoglobin changes remains uncertain, particularly with regard to their ability to reflect true hidden blood loss at the individual patient level. Clarifying the degree of agreement between hemoglobin-based estimations and established calculation methods, such as the Sehat formula, is therefore essential to define the role of hemoglobin drop in contemporary perioperative blood management.

Importantly, both perioperative hemoglobin drop and formula-based hidden blood loss estimation represent indirect approaches based on shared physiological parameters. Therefore, this study does not aim to validate one method against a reference standard, but rather to assess agreement between two imperfect estimators.

The aim of this study was to evaluate the agreement between perioperative hemoglobin drop and Sehat-derived hidden blood loss and to assess the clinical interpretability of hemoglobin-based blood loss estimation in elective primary total hip arthroplasty. Secondary aims were to quantify agreement across the full range of blood loss, to explore the influence of transfusion, cement usage, and patient-related factors, and to assess the discriminative performance of hemoglobin drop for identifying patients with high hidden blood loss.

## Methods

### Data collection and processing

This retrospective registry-based study was conducted in accordance with the STROBE guidelines (see Supplementary Material for the completed checklist) [11]. We included all elective primary THA cases performed at the University Hospital of Brandenburg/Havel from 1 January 2016 to 31 December 2023. Approval for analysis of these data had been previously granted (University of Brandenburg Ethics Committee, 292032025-BO-E-RETRO), and the present work is covered as a secondary analysis.

The present analysis was predefined as part of an overarching perioperative blood management research framework, called the *Brandenburg THA Blood Management Series* [12–14], a coherent body of work designed to advance evidence-based perioperative management in elective total hip arthroplasty.

### Study population

All adult patients undergoing elective primary THA within the study period were eligible. Exclusion criteria comprised fracture-related arthroplasty, revision procedures, hemiarthroplasty, pathological fractures, and cases with incomplete perioperative hemoglobin data. Each patient was included once. Tranexamic acid was routinely administered as part of standardized perioperative blood management protocols in elective THA. Patients with missing perioperative hemoglobin values were excluded from the respective analyses.

### Hemoglobin measurements and definition of hemoglobin drop

Preoperative hemoglobin was defined as the last value obtained within 24 h before surgery. Postoperative hemoglobin was defined as the lowest recorded value within the first 48 h after surgery. Hemoglobin drop was calculated as the absolute difference between preoperative hemoglobin and postoperative nadir hemoglobin. In cases with multiple postoperative hemoglobin measurements within the first 48 h, the lowest recorded value was used to define hemoglobin drop.

### Estimation of hidden blood loss

Hidden blood loss was calculated using the Gross–Sehat method [15, 16]. Patient blood volume was estimated according to the Nadler formula based on height, weight, and sex. Total blood loss was calculated using the Gross formula based on perioperative hematocrit changes. Hidden blood loss was subsequently derived according to the Sehat method as the difference between total blood loss and measured perioperative blood loss, including intraoperative suction volumes and transfused blood products. All values were expressed in milliliters.

### Handling of transfusions

Transfusion decisions followed a restrictive strategy and were generally considered at hemoglobin levels below 70–80 g/L or in the presence of clinical symptoms of anemia. Hemoglobin values obtained after allogeneic blood transfusion were excluded from hemoglobin drop calculations. For patients receiving transfusion before postoperative hemoglobin measurement, hemoglobin drop was calculated using the last available pre-transfusion hemoglobin value. Transfusion volumes were incorporated into Sehat-based blood loss estimation [15, 16] according to standard assumptions.

### Outcomes

Primary outcome: Agreement between hemoglobin drop and Sehat-derived hidden blood loss [15, 16]. Secondary outcomes included correlation and agreement stratified by transfusion status, cement usage, and predefined patient subgroups (e.g., sex, body mass index, and baseline hemoglobin).

### Statistical analysis

Agreement between hemoglobin drop and hidden blood loss was assessed using Bland–Altman analysis with calculation of mean bias and limits of agreement. Intraclass correlation coefficients were calculated as complementary measures of agreement. The association between hemoglobin drop and hidden blood loss was further explored using linear regression analysis. Discriminative performance of hemoglobin drop for identifying patients with high hidden blood loss (upper quartile) was evaluated using receiver operating characteristic (ROC) analysis. A sensitivity analysis was performed using hemoglobin values obtained between postoperative days 3 and 6 in the subset of patients with available data. All analyses were predefined and performed using standard statistical software. A two-sided p value < 0.05 was considered statistically significant.

## Results

### Study population and baseline characteristics

A total of 828 patients met the inclusion criteria and were included in the analysis. The mean patient age was 70.8 ± 10.3 years, and 33.2% of patients were male. Mean preoperative hemoglobin was 138.0 ± 13.7 g/L. Overall, 16.0% of patients received perioperative allogeneic blood transfusion. Anthropometric characteristics were as follows: mean body weight was 83.4 ± 18.7 kg, mean height was 167.9 ± 9.9 cm, and mean body mass index was 29.7 ± 10.4 kg/m² (Table 1). Baseline demographic characteristics are summarized in Table 1, while perioperative parameters are summarized in Table 2.

**Table 1:** Baseline demographic and clinical characteristics of patients undergoing elective primary total hip arthroplasty

Variable	Value
Age (years)	70.8 ± 10.3
Male sex (%)	33.2
Weight (kg)	83.4 ± 18.7
Height (cm)	167.9 ± 9.9
BMI (kg/m²)	29.7 ± 10.4
Preoperative hemoglobin (g/L)	138.0 ± 13.7

Values are presented as mean ± standard deviation or number (percentage)

**Table 2:** Perioperative blood loss and clinical parameters

Variable	Value
Intraoperative blood loss (mL)	529.8 ± 311.1
Total blood loss (mL)	1372.5 ± 504.5
Hidden blood loss (mL)	854.9 ± 443.0
Hemoglobin drop 48 h (g/L)	33.5 ± 12.3
Any transfusion (%)	16.0
Operative time (min)	72.0 ± 22.9

Hidden blood loss accounted for the majority of total blood loss (854.9 ± 443.0 mL vs. 529.8 ± 311.1 mL intraoperative blood loss). Values are presented as mean ± standard deviation or number (percentage)

### Perioperative blood loss and hemoglobin changes

Descriptive perioperative blood loss parameters are shown in Table 2. Mean intraoperative blood loss was 529.8 ± 311.1 mL, while mean Sehat-derived total blood loss was 1372.5 ± 504.5 mL. Mean hidden blood loss amounted to 854.9 ± 443.0 mL, indicating that the majority of perioperative blood loss was not captured intraoperatively. The mean absolute perioperative hemoglobin drop within the first 48 h was 33.5 ± 12.3 g/L, with substantial interindividual variability.

### Agreement between hemoglobin drop and Sehat-derived hidden blood loss

**Fig. 1:**
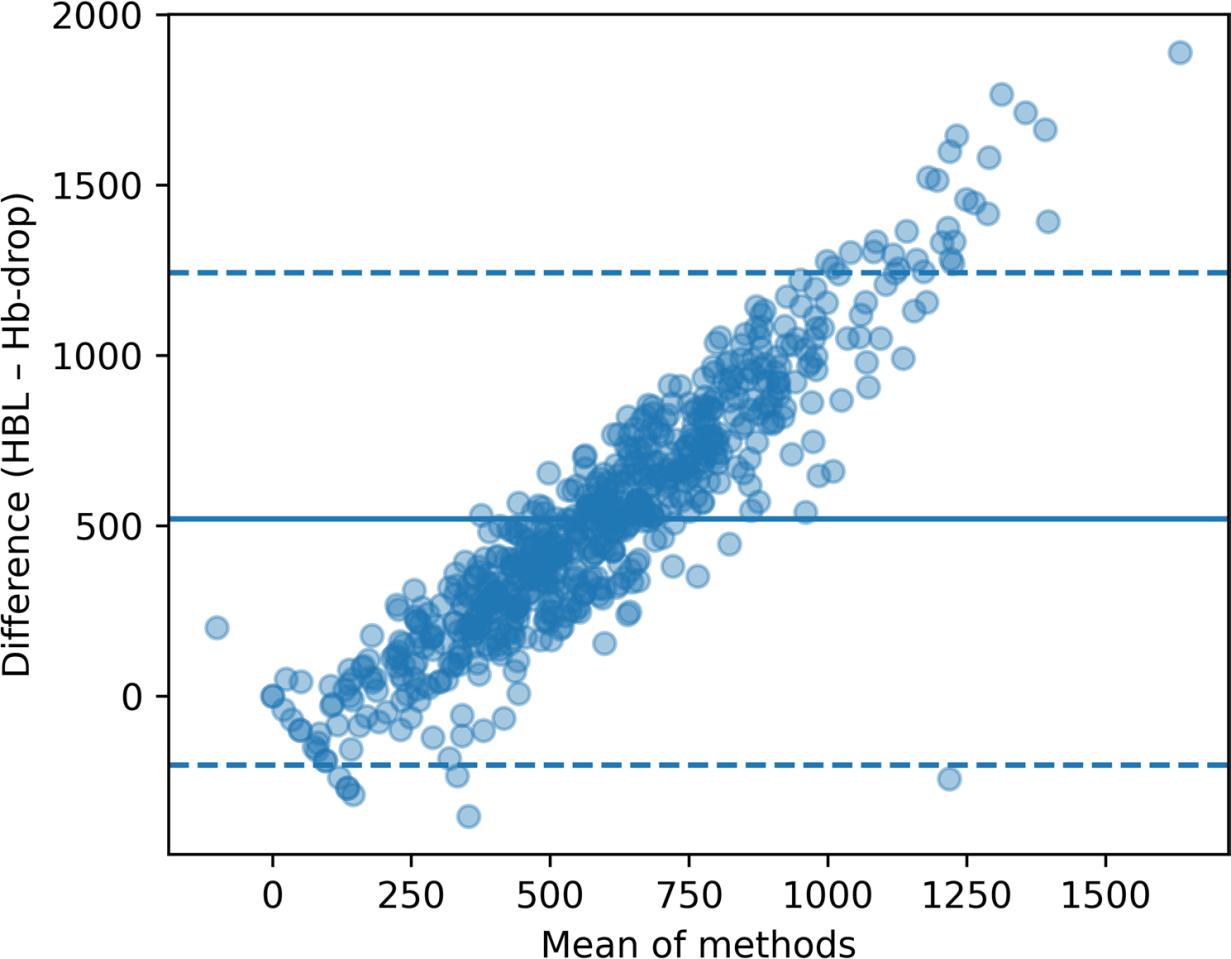
Bland–Altman plot comparing hemoglobin drop and hidden blood loss. Bland–Altman analysis illustrating the agreement between perioperative hemoglobin drop and Sehat-derived hidden blood loss. The solid line represents the mean bias, and dashed lines indicate the limits of agreement

Agreement statistics comparing perioperative hemoglobin drop with Sehat-derived hidden blood loss are summarized in Table 3. Bland–Altman analysis demonstrated a systematic bias with wide limits of agreement, indicating only moderate concordance between hemoglobin drop and calculated hidden blood loss at the individual patient level. The mean bias was 520.4 mL (95% limits of agreement − 202.2 to 1242.9 mL). The Bland–Altman plot illustrating the agreement across the full measurement range is shown in Fig. 1. Increasing dispersion was observed with higher blood loss values. A sensitivity analysis using hemoglobin values between postoperative days 3 and 6 yielded comparable results, without relevant change in agreement between hemoglobin drop and Sehat-derived hidden blood loss.

**Table 3:** Agreement statistics comparing perioperative hemoglobin drop and Sehat-derived hidden blood loss

Metric	Value
Mean bias (mL)	520.4
Limits of agreement (mL)	-202.2 to 1242.9

Bland–Altman analysis demonstrated a mean bias of 520.4 mL with wide limits of agreement (−202.2 to 1242.9 mL), indicating limited agreement at the individual patient level

### Association between hemoglobin drop and hidden blood loss

**Fig. 2:**
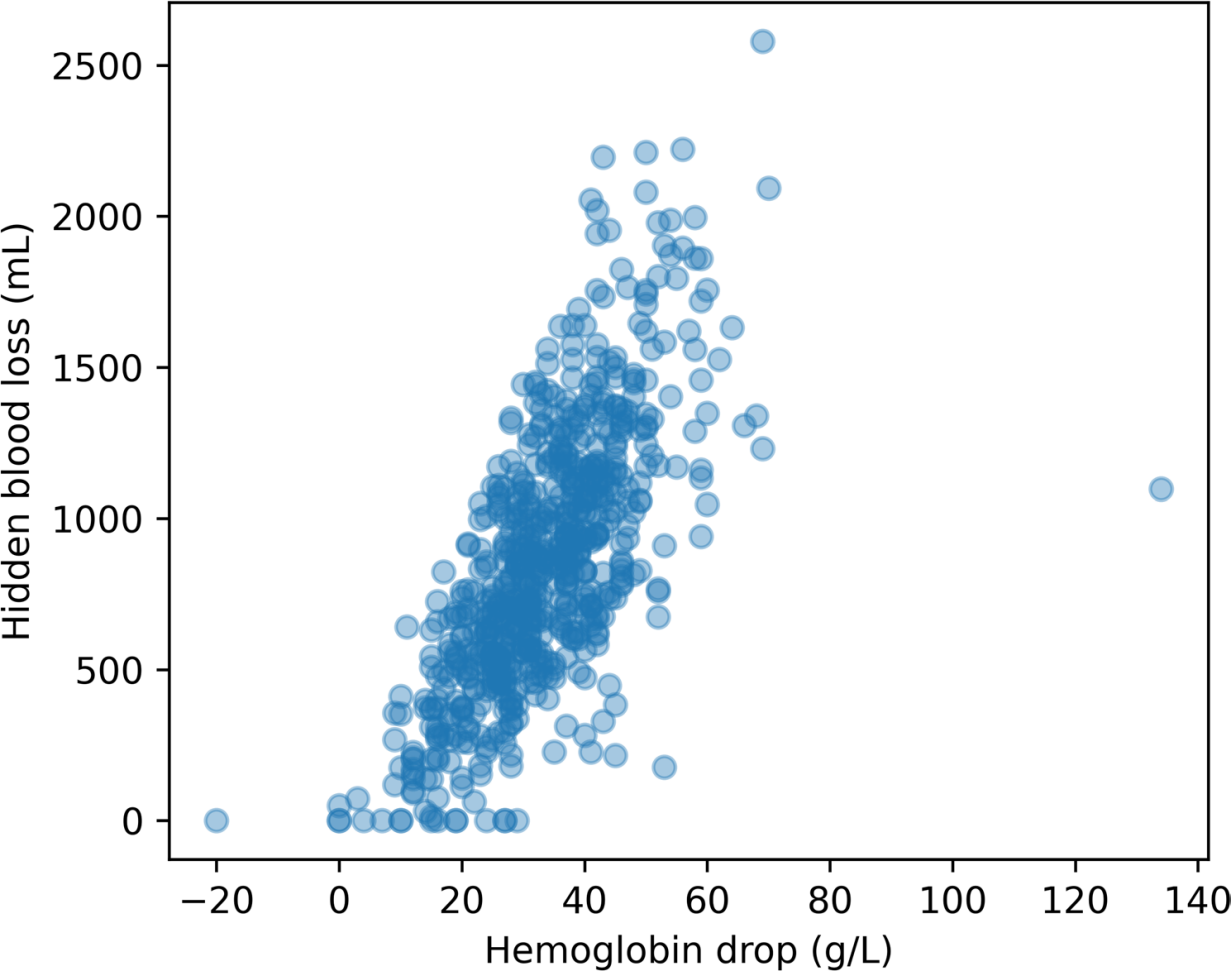
Association between hemoglobin drop and hidden blood loss. Scatterplot showing the relationship between perioperative hemoglobin drop and Sehat-derived hidden blood loss. Despite a positive association, substantial dispersion indicates limited accuracy of hemoglobin drop as a point estimator

Scatterplot analysis demonstrated a positive association between perioperative hemoglobin drop and Sehat-derived hidden blood loss (Fig. 2). However, marked dispersion around the regression line was observed, particularly at higher levels of blood loss, indicating limited precision of hemoglobin drop as a quantitative surrogate for hidden blood loss.

### Discriminative performance for high hidden blood loss

**Fig. 3:**
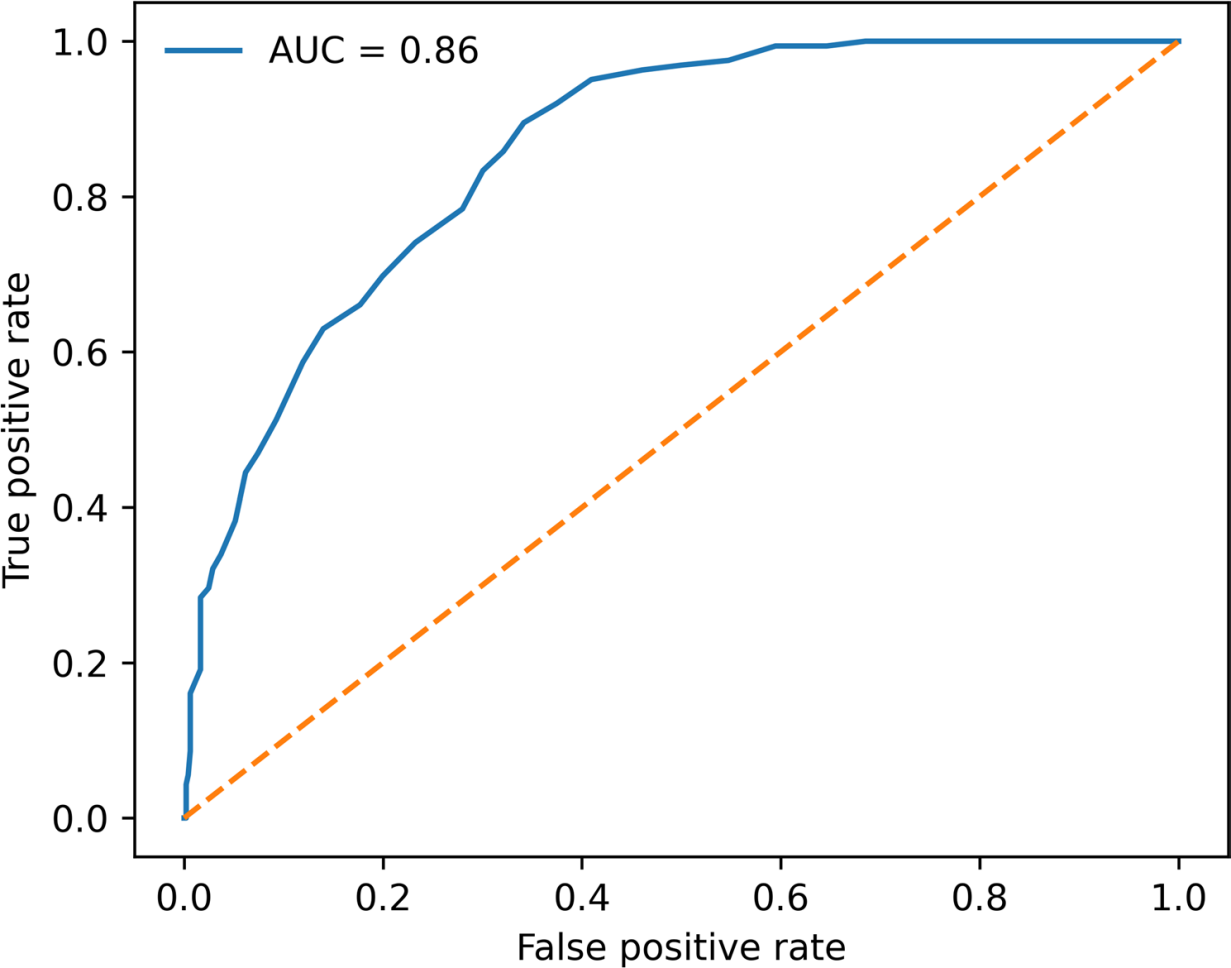
ROC curve for identification of high hidden blood loss. Receiver operating characteristic curve assessing the discriminative performance of hemoglobin drop for identifying patients with high hidden blood loss (upper quartile). Hemoglobin drop demonstrates moderate discrimination without a clearly optimal cutoff

Receiver operating characteristic analysis evaluating hemoglobin drop for identification of patients with high hidden blood loss (upper quartile) is presented in Fig. 3. Hemoglobin drop demonstrated good discriminative performance (AUC = 0.86), without a clearly optimal threshold achieving both high sensitivity and specificity.

## Discussion

This study evaluated the agreement between perioperative hemoglobin drop and Sehat-derived hidden blood loss and, importantly, the clinical interpretability of hemoglobin-based blood loss estimation in elective primary total hip arthroplasty. The principal finding is that, although hemoglobin drop correlates with calculated hidden blood loss at the population level, agreement between the two methods at the individual patient level is limited. These findings indicate that hemoglobin drop cannot be used as a reliable quantitative measure of hidden blood loss in individual patients and highlight its role as a clinically accessible but limited parameter in perioperative blood management.

### Agreement between hemoglobin drop and hidden blood loss

Bland–Altman analysis demonstrated wide limits of agreement between hemoglobin drop and Sehat-derived hidden blood loss, indicating substantial interindividual variability. While a systematic relationship was observed, the magnitude of disagreement increased at higher blood loss values, suggesting that hemoglobin drop lacks sufficient precision to reliably quantify hidden blood loss in individual patients. These findings highlight the fundamental methodological difference between a dilution-based blood loss estimate and a single-parameter laboratory change.

### Clinical interpretation of hemoglobin drop

Hemoglobin drop reflects a composite of true blood loss, perioperative hemodilution, fluid shifts, and erythrocyte redistribution and therefore cannot be interpreted as a direct measure of blood loss at the individual patient level. In contrast, the Sehat method integrates hematocrit changes with estimated blood volume and measured losses, providing a more comprehensive approximation of total and hidden blood loss. The moderate discriminative performance of hemoglobin drop for identifying patients with high hidden blood loss further supports its role as a screening or risk stratification marker rather than as a quantitative substitute for formal blood loss calculations. Importantly, the Sehat method itself represents an indirect estimation based on assumptions regarding blood volume, fluid shifts, and timing of laboratory measurements and should therefore not be interpreted as a true gold standard.

### Comparison with literature

The present findings should be interpreted within the context of our previously published work from the *Brandenburg THA Blood Management Series* [12–14]. In this series, procedure-related factors such as femoral stem design and fixation technique were shown to influence intraoperative and total blood loss, but did not independently affect transfusion risk or postoperative complications after adjustment for patient-related variables [12, 14]. In parallel, our analysis of transfusion predictors demonstrated that early postoperative hematologic parameters, particularly 48-hour hematocrit, are strongly associated with transfusion risk and may serve as clinically actionable markers [13].

Taken together, these findings provide a consistent pattern: while surgical and implant-related factors may influence measured blood loss, clinically relevant outcomes such as transfusion are predominantly driven by patient-related and physiological factors [12–14]. In this context, the present study further refines this concept by demonstrating that perioperative hemoglobin drop, although correlated with calculated blood loss at the population level, lacks sufficient precision for individual quantification and should therefore be interpreted as a qualitative rather than quantitative parameter. This reinforces the importance of integrating hemoglobin changes into a broader clinical framework rather than using them as standalone estimates of blood loss.

Furthermore, these findings are consistent with previous studies reporting substantial variability and limited precision of hemoglobin-based blood loss estimation, particularly in the presence of perioperative fluid shifts and transfusion [17]. Similar observations have been reported in both arthroplasty and other surgical settings, where hemoglobin changes were shown to correlate at the population level but lacked sufficient accuracy for individual patient-level quantification [18].

### Implications for perioperative blood management

Hemoglobin drop remains clinically useful for identifying patients at increased risk of substantial blood loss who may benefit from closer monitoring or targeted blood management strategies. Importantly, reliance on hemoglobin drop alone may lead to under- or overestimation of true blood loss, particularly in patients with pronounced hemodilution or perioperative transfusion. From a clinical perspective, hemoglobin drop should be interpreted as a qualitative indicator of perioperative blood loss rather than a quantitative measure. It may be useful for early identification of patients at risk of substantial blood loss, but should not be used in isolation to estimate actual blood loss or to guide transfusion decisions. Instead, clinical decision-making should integrate hemoglobin trends with patient-specific factors and perioperative context.

### Strengths and limitations

Strengths of this study include the large consecutive cohort, standardized perioperative laboratory assessment, and the use of agreement-based statistical methods rather than simple correlation analyses. Transfusion was explicitly accounted for in both hemoglobin-based and Sehat-derived calculations, reducing a common source of bias in blood loss studies.

Several limitations should be acknowledged. The retrospective design limits causal inference, and residual confounding cannot be excluded. Sehat-derived hidden blood loss itself represents an estimate rather than a direct measurement and may be influenced by perioperative fluid management and timing of laboratory sampling. Hemoglobin nadir was defined within the first 48 h postoperatively; later nadirs may have been missed in a subset of patients. Perioperative fluid management represents a major uncontrolled confounder in the present study, as both hemoglobin drop and Sehat-derived blood loss estimates are sensitive to hemodilution. Detailed data on fluid volumes and types were not consistently available and could therefore not be included in the analysis. Although perioperative fluid administration followed standardized institutional protocols, individual variability cannot be excluded and may have contributed to interindividual differences in hemoglobin-based estimates. Other blood products and fluid therapies were not systematically recorded and may have contributed to variability in hemoglobin-based estimates. Finally, results reflect practice at a single high-volume center and may not be fully generalizable.

## Conclusion

Perioperative hemoglobin drop shows only moderate agreement with Sehat-derived hidden blood loss in elective primary total hip arthroplasty and cannot reliably replace dilution-based blood loss estimation at the individual patient level. While hemoglobin drop may be useful for risk stratification, formal blood loss calculations remain necessary for accurate assessment of hidden blood loss in clinical practice and research.

## Supplementary Information

Supplementary Material 1.

## Data Availability

The datasets used and/or analyzed during the current study are available from the corresponding author on reasonable request.
